# Deep Learning-Based Pathology Image Analysis Enhances Magee Feature Correlation With Oncotype DX Breast Recurrence Score

**DOI:** 10.3389/fmed.2022.886763

**Published:** 2022-06-14

**Authors:** Hongxiao Li, Jigang Wang, Zaibo Li, Melad Dababneh, Fusheng Wang, Peng Zhao, Geoffrey H. Smith, George Teodoro, Meijie Li, Jun Kong, Xiaoxian Li

**Affiliations:** ^1^Department of Mathematics and Statistics, Georgia State University, Atlanta, GA, United States; ^2^Institute of Biomedical Engineering, Chinese Academy of Medical Sciences and Peking Union Medical College, Tianjin, China; ^3^Department of Pathology, The Affiliated Hospital of Qingdao University, Qingdao, China; ^4^Department of Pathology and Laboratory Medicine, Emory University, Atlanta, GA, United States; ^5^Department of Pathology, The Ohio State University, Columbus, OH, United States; ^6^Department of Computer Science, Stony Brook University, Stony Brook, NY, United States; ^7^Department of Computer Science, Federal University of Minas Gerais, Belo Horizonte, Brazil; ^8^Department of Computer Science, Georgia State University, Atlanta, GA, United States; ^9^Department of Computer Science, Emory University, Atlanta, GA, United States

**Keywords:** deep learning-based algorithm, digital pathology, Oncotype DX score, ER+ breast cancer, Magee equation

## Abstract

**Background:**

Oncotype DX Recurrence Score (RS) has been widely used to predict chemotherapy benefits in patients with estrogen receptor-positive breast cancer. Studies showed that the features used in Magee equations correlate with RS. We aimed to examine whether deep learning (DL)-based histology image analyses can enhance such correlations.

**Methods:**

We retrieved 382 cases with RS diagnosed between 2011 and 2015 from the Emory University and the Ohio State University. All patients received surgery. DL models were developed to detect nuclei of tumor cells and tumor-infiltrating lymphocytes (TILs) and segment tumor cell nuclei in hematoxylin and eosin (H&E) stained histopathology whole slide images (WSIs). Based on the DL-based analysis, we derived image features from WSIs, such as tumor cell number, TIL number variance, and nuclear grades. The entire patient cohorts were divided into one training set (125 cases) and two validation sets (82 and 175 cases) based on the data sources and WSI resolutions. The training set was used to train the linear regression models to predict RS. For prediction performance comparison, we used independent variables from Magee features alone or the combination of WSI-derived image and Magee features.

**Results:**

The Pearson’s correlation coefficients between the actual RS and predicted RS by DL-based analysis were 0.7058 (*p*-value = 1.32 × 10^–13^) and 0.5041 (*p*-value = 1.15 × 10^–12^) for the validation sets 1 and 2, respectively. The adjusted *R*^2^ values using Magee features alone are 0.3442 and 0.2167 in the two validation sets, respectively. In contrast, the adjusted *R*^2^ values were enhanced to 0.4431 and 0.2182 when WSI-derived imaging features were jointly used with Magee features.

**Conclusion:**

Our results suggest that DL-based digital pathological features can enhance Magee feature correlation with RS.

## Background

Breast cancer is the most common cancer in women in the United States. Breast cancers are clinically classified by the expression of estrogen receptor (ER), progesterone receptor (PR), and human epidermal growth factor receptor 2 (HER2) gene amplification as ER+/ HER2-, HER2+, and triple-negative (ER-/PR-/HER2-) subtypes. Each subtype has unique tumor biology, treatment options, and prognosis (1–7). Approximately 70% of the breast cancers are ER+/HER2-. Patients with HER2+ and triple-negative breast cancer are generally treated with chemotherapy. However, only a portion of the patient with ER+/HER2- breast cancer benefit from chemotherapy (6, 8–10). Whether patients with ER+/HER2- breast cancer benefit from chemotherapy depends on such clinicopathological features as tumor grade and size, tumor cell proliferation, staging, and molecular profile biomarkers. Before the clinical validation of molecular biomarkers, most patients with high-risk ER+/HER2- breast cancer were treated with chemotherapy (11, 12). Oncotype DX Recurrence Score (RS) uses a 21-gene expression profile to predict prognosis and determine the benefit of chemotherapy in patients with ER+/HER2- breast cancer (13–15). The predictive value of RS was validated by large prospective trials and prospective-retrospective studies (14, 15).

The TAILORx trial has validated RS predictive value for patients with ER+/HER2- and lymph node (LN) negative breast cancer. The first publication in 2015 from the TAILORx trial showed that patients with an RS of 0–10 had an excellent prognosis and were highly unlikely to benefit from chemotherapy (16). The second publication from the TAILORx trial showed patients > 50 years old and some young patients (≤50 years old) with a medium RS could be spared from chemotherapy (13). Recent results from the RxPONDER study showed that RS could also predict chemotherapy benefits in patients with ER+/HER2- and 1–3 LN+ breast cancer (17).

Magee equations use routinely available clinicopathological parameters (or Magee features) and are strongly associated with RS (18–20). Furthermore, machine learning-based histology analysis has been shown to correlate with prognosis and behaviors in diseases, including breast cancer (21–26). Therefore, the aim of this study was to examine whether histopathological features from whole slide images (WSIs), when used with Magee features, would improve the RS prediction. Due to the overwhelming gigapixel scale of histopathology WSIs and artifacts in histopathology WSIs, it is technically challenging to extract imaging features with predictive value. Recent applications of artificial intelligence techniques in a large number of biomedical investigations (27–29) show that the deep learning (DL) model can be a potential solution to this challenge. In this study, a DL-based pipeline for WSI analysis was developed to (1) detect the tumor cell nuclei and tumor-infiltrating lymphocyte (TIL) nuclei for cell density evaluation and (2) segment tumor cell nuclei for nuclear-grade assessment. Such large-scale detection and segmentation analyses enable automatic image feature extraction from gigapixel WSIs. We examined whether the image features could enhance the correlation of Magee features with RS.

## Materials and Methods

### Datasets and Clinicopathological Information

Three independent patient cohorts with available RS were collected from two institutions and divided into training and validation sets based on the data sources and WSI resolutions. RS was defined as low (≤15), intermediate (16–25), and high (26–100) according to the results from the TAILORx trial (30). ER, PR, and HER2 interpretations were based on the updated ASCO/CAP recommendations (31, 32). All patients received surgery.

Training set: A total of 125 cases of ER+/HER2-/LN- breast cancer with RS diagnosed from 2011 to 2015 were collected from the Ohio State University. The RS ranged from 0 to 40. Among these 125 cases, 53, 59, and 13 cases had low scores, intermediate scores, and high scores, respectively.

Validation set 1: A total of 82 cases of ER+/HER2-/LN- breast cancer with RS diagnosed from 2012 to 2014 were retrieved from the Emory University. The RS ranged from 0 to 52. Among 82 cases, 40, 15, and 27 cases had low scores, intermediate scores, and high scores, respectively.

Validation set 2: Additional 175 cases of ER+/HER2-/LN- breast cancer with RS diagnosed from 2012 to 2014 were retrieved from the Emory University. The RS in this dataset ranged from 0 to 100. Among 175 cases, 68, 73, and 34 were low-, intermediate-, and high-score cases, respectively.

All three datasets included age at diagnosis, ER and PR IHC staining percentage (0–100) and intensity (1, 2, and 3), HER2 amplification by IHC and FISH (negative and equivocal), Nottingham tumor grade, and tumor size. Additional features retrieved for validation sets 1 and 2 included Ki-67 score, stage, chemotherapy, radiation therapy, overall survival (OS), disease-free survival (DFS), and distant metastasis (metastasis other than axillary LN metastasis). One representative tumor hematoxylin and eosin (H&E) stained WSI from each case in the training set and validation set 1 was scanned at 40 × magnification and validation set 2 at 20 × with an Aperio AT2 scanner.

The clinicopathological information of these three datasets is summarized in Table 1. The ER and PR expressions for all three cohort datasets were evaluated with an H-score (percentage × intensity). This study was approved by the Institutional Review Board at the Emory University and the Ohio State University.

**TABLE 1:** Clinicopathological information of the three datasets.

	Training set	Validation set 1	Validation set 2
**Nottingham grade (case number)**			
1	33 (26.4%)	31 (37.8%)	64 (36.6%)
2	75 (60.0%)	39 (47.6%)	92 (52.6%)
3	17 (13.6%)	12 (14.6%)	19 (10.8%)
**ER intensity (case number)**			
0	0 (0.0%)	0 (0.0%)	0 (0.0%)
1	0 (0.0%)	2 (2.4%)	3 (1.7%)
2	9 (7.2%)	9 (11.0%)	33 (18.9%)
3	116 (92.8%)	71 (86.6%)	139 (79.4%)
**ER percentage**			
Mean	94.21	89.63	87.59
Range	40–100	5–100	10–100
**PR intensity (case number)**			
0	8 (6.4%)	17 (20.7%)	19 (10.9%)
1	3 (2.4%)	3 (3.7%)	5 (2.9%)
2	30 (24.0%)	16 (19.5%)	38 (21.7%)
3	84 (67.2%)	46 (56.1%)	113 (64.6%)
**PR percentage**			
Mean	66.29	55.15	62.3
Range	0–100	0–100	0–100
**HER2 (case number)**			
Negative	123 (98.4%)	81 (98.8%)	173 (98.9%)
Equivocal positive	2 (1.6%)	1 (1.2%)	2 (1.1%)

**Ki-67 score**	**(Not available)**		**(105/175 cases available)**

Mean	N/A	24.26	29.09
Range	N/A	1–100	1–91
**Tumor size (cm)**			
Mean	2.19	1.81	1.64
Range	0.4–7.8	0.5–5.3	0.3–7.1
**Age (year)**			
Mean	58.00	60.29	56.97
Range	32–82	31–81	30–91
**Oncotype DX RS**			
Mean	16.62	19.15	18.93
Range	0–40	0–52	0–100

**Real chemotherapy (case number)**		**(80/82 cases available)**	**(168/175 cases available)**

Yes	N/A	24 (29.3%)	48 (27.4%)
No	N/A	56 (68.3%)	120 (68.6%)
OS (months)			
Mean	N/A	32.80	81.45
Range	N/A	1–250	0–272

**DFS (months)**		**(4/82 cases available)**	**(9/175 cases available)**

Mean	N/A	75.25	64.74
Range	N/A	3–151	12–174

**Real radiation therapy (case number)**		**(80/82 cases available)**	**(168/175 cases available)**

Yes	N/A	49 (59.8%)	99 (56.6%)
No	N/A	31 (37.8%)	69 (39.4%)
**Stage (case number)**			
1	91 (72.8%)	55 (67.1%)	122 (69.7%)
2	29 (23.2%)	26 (31.7%)	50 (28.6%)
3	5 (4.0%)	1 (1.2%)	3 (1.7%)

**Distant metastasis (case number)**		**(81/82 cases available)**	**(169/175 cases available)**

Yes	N/A	2 (2.4%)	6 (3.4%)
No	N/A	79 (97.6%)	163 (96.6%)

### Data Preprocessing

**FIGURE 1:**
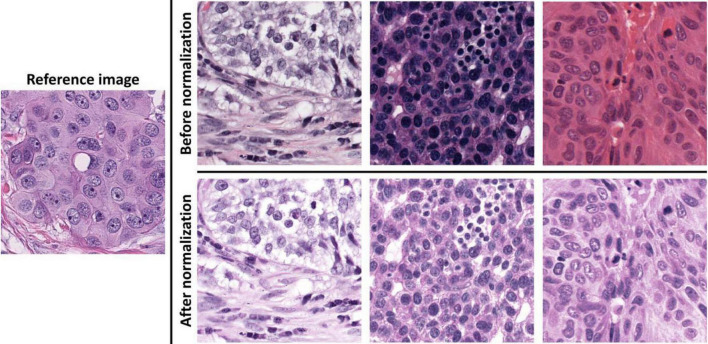
Demonstrations of image color normalization. With the learned color and brightness information from the reference image on the left, three randomly selected images before and after color normalizations are presented on the top and bottom rows on the right.

Image normalization: As 40 × images have a higher resolution for annotations, we chose the 40 × for data analysis. After linearly resizing with a scaling factor of two along the image width and height directions, all images in validation set 2 had the same magnification of 40 × as training set and validation set 1. We also used the sparse non-negative matrix factorization-based color transfer method (33) to normalize the image color styles in all three cohort datasets (Figure 1).

Data preprocessing for DL training: Although we had three datasets for RS prediction analysis, we used two independent image datasets for cell detection and segmentation training, one from our lab and the other from the public MoNuSeg-2018 dataset. We collected 797 images with tumor nuclei point annotations, 500 images with TIL point annotations, and 26 images with annotations of tumor nuclei contours from the independent dataset. All the annotations were produced and confirmed by the pathologists (Supplementary Figure 1). Two pathologists made the annotations with Aperio ImageScope and GIMP. Additionally, 30 H&E images from the public MoNuSeg-2018 dataset were used in the segmentation dataset. They had annotations of cell nucleus contours (Supplementary Figure 1C). Each DL dataset was randomly divided into training, validation, and testing groups with an approximate proportion of 70:15:15.

### Deep Learning Model

**FIGURE 2:**
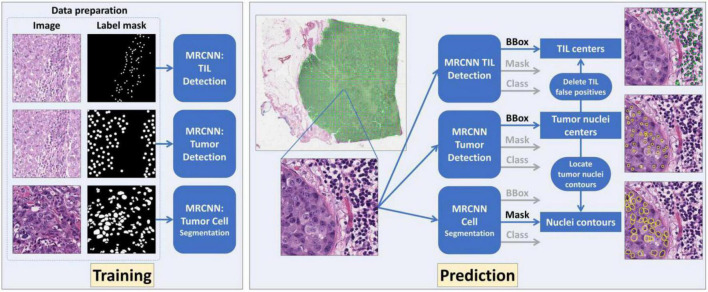
The overall schema of the developed deep learning (DL)-based whole slide image (WSI) processing pipeline is presented. Three DL models were established and trained for tumor cell detection, tumor-infiltrating lymphocyte (TIL) detection, and tumor cell segmentation, respectively. The tumor cell detection results were used to remove TIL false positive and retain nuclei contours for tumor cell segmentation.

For detection, classification, and segmentation analyses, we used the Mask R-CNN (MRCNN) (34) to construct the image processing models in this project. MRCNN was extended from Faster R-CNN (35) that was in turn developed based on Fast R-CNN (36). The overall schema of the developed WSI image processing pipeline is presented in Figure 2. The DL MRCNN pipeline was constructed with library TensorFlow and Keras. The image processing module contained three MRCNN models specifically for tumor cell detection, TIL detection, and tumor nucleus segmentation, respectively. Image tiles with tissue were extracted from WSIs by thresholding the “Saturation” channel of the HSV color space with the threshold set to 30. Each image tile was then analyzed by three MRCNN models separately. The center of each bounding box is considered the center of a detected cell of interest. The segmentation branch in the MRCNN model produced nucleus contours. Since the tumor cell detection had superior performance, the detected tumor cells were used to exclude the TIL and tumor nucleus false positive. All computational analyses were executed on a computational server with two CPUs of 22 2.10 GHz cores each, 192 GB memory, and six Nvidia GeForce RTX 2080 Ti GPUs with 11 GB memory each.

### Linear Regression Model Incorporating Deep Learning-Based Imaging Features and Magee Equation Variables

We partitioned each WSI into image tiles with a size of 1,024 × 1,024 by pixels to identify tissue regions of high tumor cell density with the DL-based processing pipeline. The top ten image tiles with the highest tumor cell density in each WSI were selected for feature extraction. To generate interpretable models, we chose to select image features of interpretability instead of hidden or intermediate features by machine learning algorithms. Since tumor cells and TILs were reported high correlation with the prognosis or recurrence (37, 38), we extracted three tile-wise features from each image tile, including (1) the tumor cell number, (2) the TIL number, and (3) the tumor cell percentage. Additionally, nuclear grade and TIL number variance were extracted from the ten image tiles collectively. The nuclear grade of each tumor cell was determined by comparing the tumor nuclei size with the adjacent TIL nuclei size. The TIL nuclei size was 304.7 in pixels averaged from representative TILs selected by pathologists. Nuclear grade 1 was defined when the ratio of tumor nucleus size to TIL nucleus size was 1–2.5. Nuclear grade 2 was made when such a ratio was 2.5–3.5. Nuclear grade 3 was made when such a ratio was > 3.5 (Supplementary Figure 2). Tumor cell nuclear grades from the ten image tiles were collected and aggregated to a final nuclear grade by the following rules: (1) if ≥ 10% of the tumor cells had nuclear grade 3, the aggregated nuclear grade was 3; (2) if ≥ 10% of the tumor cells had nuclear grade 2 and rule (1) did not hold, the aggregated nuclear grade was 2; (3) if ≥ 10% of the tumor cells had nuclear grade 1 and neither rule (1) nor (2) held, the final nuclear grade was 1. The image feature of TIL number variance was also computed from the top ten image tiles by cell density as follows:


$$V=\frac{\sum_{i=1}^{10}\left(n_{i}-\bar{n}\right)}{10-1}$$

where *V* is the TIL number variance; *n_i_* represents the TIL number in the *i*-th image tile; $\bar{n}$ is the average TIL number from the ten image tiles. In total, there were 32 image features extracted from each WSI.

A linear regression model was used to correlate with RS. In the regression model, the dependent variable was the RS, while imaging features and Magee features were independent variables. To retain features with high predictive value, we selected features by both domain knowledge and statistical analysis. The independent variables in Magee equations are as follows (39). Magee equation 1 includes Nottingham score, ER and PR H-scores, HER2, tumor size (cm), and Ki67 index; Magee equation 2 includes Nottingham score, ER and PR H-scores, HER2, and tumor size (cm); Magee equation 3 includes ER and PR H-scores, HER2, and Ki67 index. As the feature “HER2” is categorical with two possible values, i.e., “Negative” and “Equivocal,” we used one dummy variable, “HER2_Equivocal,” to represent “HER2” in the regression models. We focused on Magee equation 2 as the Ki-67 index information was missing for more than half samples (195/382, 51.0%) in our datasets. Additionally, the tile-wise features from the first *x* out of the ten image tiles (*x* = 1, 2, …, 10), i.e., the tumor cell number, TIL number, and tumor cell percentage, were used jointly. The feature selection was completed in the training set. Various feature combinations were used to construct the linear regression models. The adjusted coefficient of determination *R*^2^ was used to assess the combinations’ correlation with RS. The feature combination with the highest adjusted *R*^2^ was selected for the final model.

## Results

### Validated Deep Learning Models Accurately Identified Tumor Nuclei, Tumor-Infiltrating Lymphocyte Nuclei, and Tumor Cell Nuclear Grade

A total of 7,609 annotated tumor nuclei from 120 testing images and 4,000 annotated TILs from 75 testing images were collected to validate the MRCNN model for tumor nuclei and TIL detection. The trained models correctly detected 6,101 (80.2%) tumor nuclei and 3,304 (82.6%) TILs. Multiple metrics were used for performance assessments, including precision, recall, F1-score, true positive number, false-positive number, and false-negative number. The metrics of precision, recall, and F1-score were defined as follows.


$$P\,r\,e\,c\,i\,s\,i\,o\,n=\frac{T\,P}{T\,P+F\,P}$$

$$R\,e\,c\,a\,l\,l=\frac{T\,P}{T\,P+F\,N}$$

$$F\,1\,s\,c\,o\,r\,e=2\times \frac{P\,r\,e\,c\,i\,s\,i\,o\,n\times R\,e\,c\,a\,l\,l}{P\,r\,e\,c\,i\,s\,i\,o\,n+R\,e\,c\,a\,l\,l}$$

where *TP*, *FP*, and *FN* represent the number of true positive, false-positive, and false-negative samples, respectively. The true positive samples were correctly detected samples. The false-positive samples were cells erroneously detected. Finally, the false-negative samples were missed ground truths from pathologists. The MRCNN models for the tumor nuclei and TIL detection achieved 0.7765 and 0.7171 for the F1-score, 0.7528 and 0.6337 for the precision, and 0.8018 and 0.826 for the recall, respectively.

**FIGURE 3:**
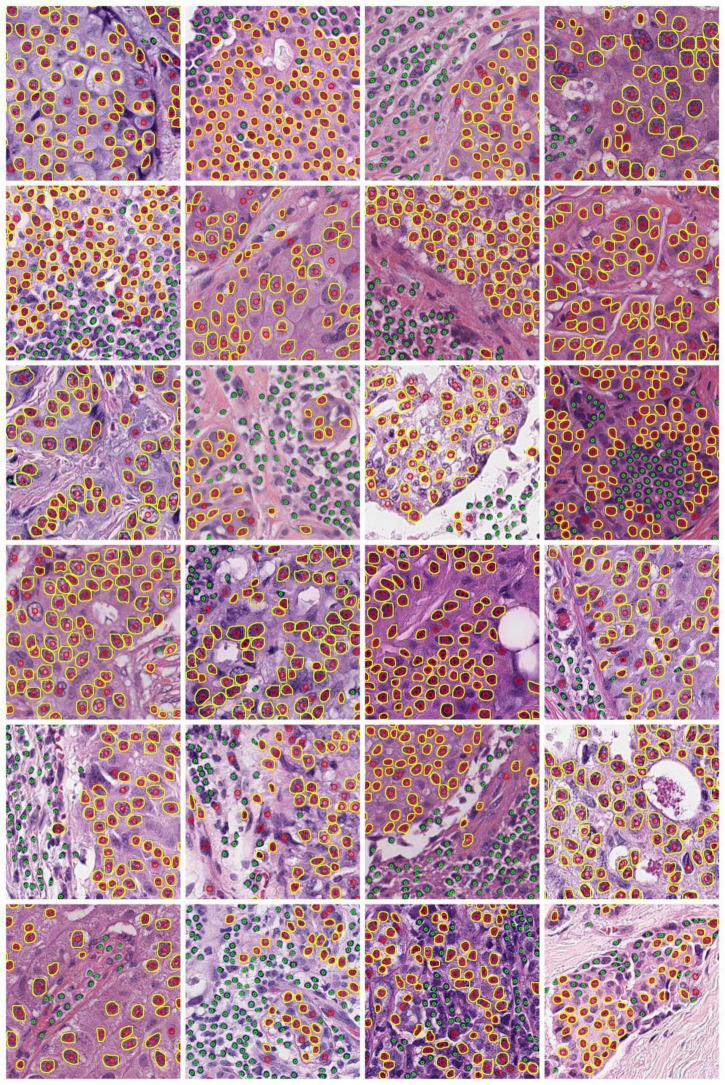
Demonstration of representative cell detection and segmentation results from DL models. Detected TIL and tumor nuclei are indicated by green and red circles, respectively. The predicted contours of tumor nuclei are indicated in yellow.

The Hausdorff distance (HD) was used to measure the tumor nucleus contour concordance between the ground truths from pathologists and predictions using the DL process (Supplementary Figure 3). The metric of intersection over union (IOU) was used to match the ground truth to predicted contours. When IOU was greater than or equal to a cutoff value *K*, the ground truth and predicted nucleus contours were considered as a matched pair. When there was more than one prediction matching the same ground truth, the prediction with the largest IOU was retained for the match. When one prediction was matched to more than one ground truth, the prediction was assigned to the first matched ground truth. The cutoff value *K* was set as 0.1, 0.2, 0.3, 0.4, 0.5, 0.6, 0.7, 0.8, and 0.9, respectively. We computed the mean equivalent nuclei diameter for each nuclear grade. The mean equivalent diameters for nuclear grades 1, 2, and 3 were 26.30, 34.29, and 48.67 pixels, respectively. We present the mean HD between matched pairs and the ratio of mean HD to the mean equivalent diameter of tumor nuclei in Table 2. Representative cell detection and segmentation results from the DL models are shown in Figure 3.

**TABLE 2:** Performance of the Mask R-CNN (MRCNN) model for tumor nucleus segmentation.

IOU cutoff value	Mean HD for G1 (pixels) and ratio %	Mean HD at G2 (pixels) and ratio %	Mean HD at G3 (pixels) and ratio %
0.1	5.07 (19.28%)	6.29 (18.33%)	11.48 (23.59%)
0.2	5.05 (19.21%)	6.29 (18.33%)	11.30 (23.23%)
0.3	5.03 (19.14%)	6.29 (18.33%)	10.94 (22.48%)
0.4	4.97 (18.88%)	6.15 (17.94%)	10.50 (21.58%)
0.5	4.70 (17.89%)	5.84 (17.04%)	9.10 (18.69%)
0.6	4.32 (16.44%)	5.63 (16.41%)	7.77 (15.97%)
0.7	3.85 (14.65%)	4.94 (14.42%)	6.21 (12.77%)
0.8	3.13 (11.91%)	4.04 (11.77%)	5.14 (10.56%)
0.9	2.24 (8.53%)	2.34 (6.84%)	3.42 (7.03%)

*The mean Hausdorff distances (HDs) of the matched ground truth and predicted contours of tumor nuclei at nuclear grades G1, G2, and G3 were computed with different intersection over union (IOU) cutoff values. For each grade, we computed the mean equivalent diameter. Additionally, we computed the ratio of mean HD to the mean equivalent diameter in percentage for each grade. The resulting mean HD and the ratio% are presented for each nuclear grade and each IOU cutoff value.*

### The Deep Learning-Based Analysis Enhances the Correlation Between Features in Magee Equation 2 and Recurrence Score

**FIGURE 4:**
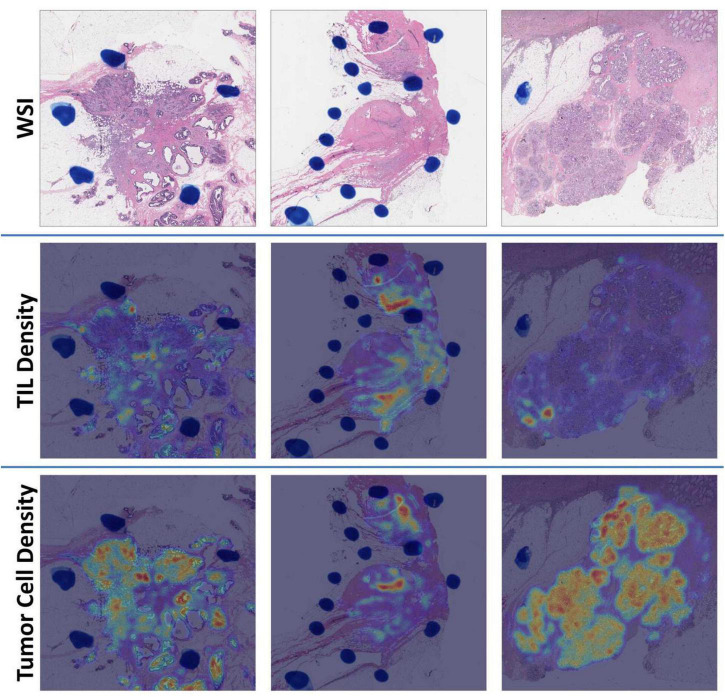
Demonstration of WSI density maps from (left) the low [Oncotype DX Recurrence Score (RS) = 3], (middle) intermediate (RS = 19), and (right) high (RS = 39) RS group. For each group, we present (top) a WSI, (middle) a TIL density map, and (bottom) a tumor cell density map, respectively.

We detected an overwhelmingly large number of cells in each WSI (Supplementary Table 1). With detection results from image tiles, tumor cell and TIL density distributions were estimated and represented as density maps (Figure 4). The top ten image tiles of each WSI were selected based on the tumor cell density.

Eight variables from the training set included Nottingham grade, ER and PR H-score HER2 status, tumor size (cm), tumor cell number in the densest tile, TIL number variance, and tumor nuclear grade (Table 3). The first five variables were from Magee equation 2, while the last three variables were DL-based image features derived from WSIs. We established a regression model with these selected features from the training set and applied the model to validation sets 1 and 2 for RS correlation.

**TABLE 3:** Summary of independent variables from training set, validation set 1, and validation set 2 for the regression model.

	Training set	Validation set 1	Validation set 2
**Nottingham grade (case number)**			
1	33	31	64
2	75	39	92
3	17	12	19
**ER H-score**			
Mean	277.14	262.18	246.03
Range	80–300	5–300	19–300
**PR H-score**			
Mean	188.07	156.07	174.47
Range	0–300	0–300	0–300
**HER2 (case number)**			
Negative	123	81	173
Equivocal positive	2	1	2
**Tumor size (cm)**			
Mean	2.19	1.81	1.64
Range	0.4–7.8	0.5–5.3	0.3–7.1
**Tumor cell number in the densest tile**			
Mean	346.13	316.23	262.55
Range	140–612	112–531	58–567
**TIL number variance**			
Mean	714.95	792.67	331.42
Range	2.49–8,227.6	4.68–10,850.01	1.17–5,849.39
**Tumor nuclear grade (case number)**			
1	1	3	9
2	81	50	102
3	43	29	64

We divided cases into low, intermediate, and high RS categories with the stratification rules from the TAILORx study (30). The concordances between the RS and our model were 56.10% and 68.0% for validation sets 1 and 2, respectively (Table 4). Additionally, the one-step discordance rates for validation sets 1 and 2 were 39.02% and 48.0%, respectively. The Pearson’s correlation coefficients between the RS and our model were 0.7058 (*p*-value = 1.32 × 10^–13^) and 0.5041 (*p*-value = 1.15 × 10^–12^) for validation sets 1 and 2, respectively. The tumor and TIL density maps from validation sets 1 and 2 are illustrated in Supplementary Figures 4, 5.

**TABLE 4:** Oncotype DX Recurrence Score (RS) group confusion matrix for validation sets 1 and 2.

	Validation set 1				Validation set 2			
	Predict high	Predict middle	Predict low	Total	Predict high	Predict middle	Predict low	Total
GT high	11	12	4	27	7	25	5	37
GT middle	0	8	7	15	1	43	26	70
GT low	0	13	27	40	0	32	36	68
Total	11	33	38	82	8	100	67	175

*The “low,” “middle,” and “high” RS levels are determined by the RS cutoff values of 16 and 25. Several summary statistics for validation sets 1 and 2 are concordance: 46/82 (56.10%) and 119/175 (68.0%); one-step discordance: 32/82 (39.02%) and 84/175 (48.0%); two-step discordance: 4/82 (4.88%) and 5/175 (2.86%); Pearson’s correlation coefficient: 0.7058 (p-value = 1.32 × 10^–13^) and 0.5041 (p-value = 1.15 × 10^–12^). GT represents ground truth.*

The performance of the model correlation with RS was further evaluated by *R*^2^ and adjusted *R*^2^ (Table 5). When the image features were integrated with features in Magee equation 2, the adjusted *R*^2^ value increased from 0.3442 (*p*-value = 5.17 × 10^–10^) to 0.4431 (*p*-value = 1.32 × 10^–13^) in validation set 1 and from 0.2167 (*p*-value = 6.52 × 10^–12^) to 0.2182 (*p*-value = 1.15 × 10^–12^) in validation set 2. Similarly, the *R*^2^ increased from 0.3846 to 0.4981 in validation set 1 and from 0.2392 to 0.2541 in validation set 2. Additionally, we demonstrated the adjusted *R*^2^ and *R*^2^ of the linear regression model that was constructed only with the image features. The resulting adjusted *R*^2^ and *R*^2^ are 0.3048 (*p*-value = 1.61 × 10^–8^) and 0.3306 (*p*-value = 1.61 × 10^–8^) for validation set 1 and 0.0139 (*p*-value = 0.0199) and 0.0309 (*p*-value = 0.0199) for validation set 2, respectively. It is noted that the image features perform much worse than Magee features in validation set 2. Such performance degradation can be related to the fact that images in validation set 2 were originally scanned at 20 × and later computationally scaled to 40 × magnification. The inconsistency in the original image magnification can contribute to a significant error in the following analyses, leading to a worse prediction result.

**TABLE 5:** Prediction performance of the regression model trained on the training set.

		Validation set 1	Validation set 2
Adjusted *R*^2^	Magee2 features	0.3442 (*p*-value = 5.17 × 10^–10^)	0.2167 (*p*-value = 6.52 × 10^–12^)
	Image features	0.3048 (*p*-value = 1.61 × 10^–8^)	0.0139 (*p*-value = 0.0199)
	Image + Magee2 features	**0.4431** (*p*-value = 1.32 × 10^–13^)	**0.2182** (*p*-value = 1.15 × 10^–12^)
*R* ^2^	Magee2 features	0.3846 (*p*-value = 5.17 × 10^–10^)	0.2392 (*p*-value = 6.52 × 10^–12^)
	Image features	0.3306 (*p*-value = 1.61 × 10^–8^)	0.0309 (*p*-value = 0.0199)
	Image + Magee2 features	**0.4981** (*p*-value = 1.32 × 10^–13^)	**0.2541** (*p*-value = 1.15 × 10^–12^)

*The bold values emphasize the greatest value of each metric in the two validation sets.*

**FIGURE 5:**
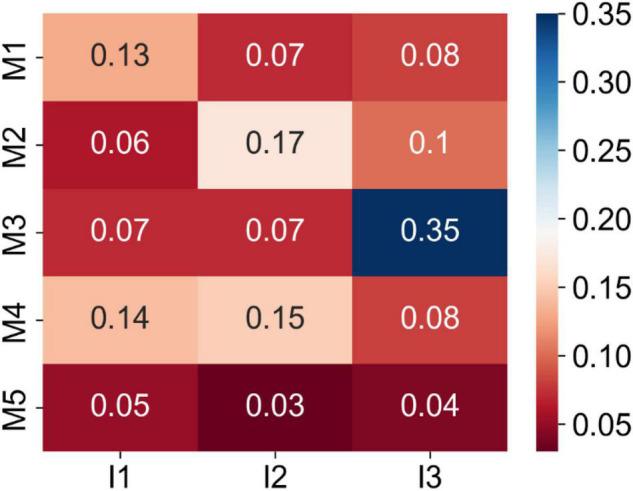
Matrix of the absolute Pearson correlation coefficients between the Magee and image features from the training set. Five Magee features M1-5 are ER H-score, PR H-score, Nottingham score, tumor size, and HER2, respectively. Three image features I1-3 are TIL number variance, tumor cell number in the densest tile, and tumor nuclear grade, respectively.

To investigate the correlations between Magee and image-derived features, we computed their pair-wise absolute Pearson correlation coefficients. As shown in Figure 5, the largest correlation coefficient of 0.35 was found by the Nottingham score and tumor nuclear grade. Five Magee and image feature pairs present correlation coefficients close to 0.1. All remaining 9 pairs present correlation coefficients less than 0.1. Such weak correlations indicate the complementary prediction value by the image features for RS prediction enhancement.

**FIGURE 6:**
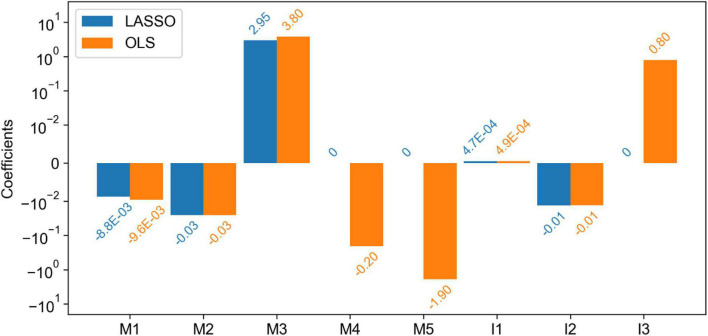
Comparison of the coefficients of features (both Magee and imaging) in the linear regression models trained by least absolute shrinkage and selection operator (LASSO) and Ordinary Least Squares (OLS). Five Magee features M1-5 are ER H-score, PR H-score, Nottingham score, tumor size, and HER2, respectively. Three image features I1-3 are TIL number variance, tumor cell number in the densest tile, and tumor nuclear grade, respectively.

For further correlation analyses between Magee and image features, we applied the least absolute shrinkage and selection operator (LASSO) regression method to our data and compared the resulting feature coefficients with those in the model trained by Ordinary Least Squares (OLS). The comparison results are presented in Figure 6. As LASSO includes an L1-norm regularizer, it penalizes the excessive feature inclusion and reduces uninformative feature coefficients to zero. From Figure 6, the non-zero feature coefficients from the two models trained by LASSO and OLS present similar values. Coefficients of only three features (i.e., tumor size, HER2, and tumor nuclear grade) were reduced to zero by LASSO. The only removed image feature by LASSO is tumor nuclear grade that presents an absolute Pearson correlation coefficient of 0.35 with the Nottingham score.

### Analyses of Cases With Discrepant Risk Scores Between Recurrence Score and Deep Learning-Based Prediction

We analyzed the cases with discordant risk categories by RS and our model (Table 6). There were totally 54 discordant cases in validation sets 1 and 2. Among these 54 cases, 40 were recommended to have chemotherapy by RS but not by our DL-based model; of these 40 cases, 28 received chemotherapy.

**TABLE 6:** Confusion matrix of the chemotherapy recommendations by RS and predicted RS for validation sets 1 and 2.

	Predicted RS No	Predicted RS Yes	Total
RS No	166	14	180
RS Yes	40	37	77
Total	206	51	257

In total, 14 cases were not recommended to have chemotherapy by RS, while our DL-based model did; of these 14 cases, 2 received chemotherapy. The chemotherapy recommendation based on RS and our DL model was determined by the suggested rules from the TAILORx study. Overall, none of these 54 discordant cases developed recurrence regardless of whether received chemotherapy, indicating that the role of chemotherapy in these discordant cases was not clear.

## Discussion

Multiple studies have demonstrated the correlations between clinicopathological features and RS. Some used regression models to predict the RS directly from the clinicopathological features (20, 39–43), while others used classifiers to predict the RS risk categories (44–53). Additionally, a few studies have shown that the tumor imaging features from mammographic and sonographic imaging (54) and MRI (55, 56) are associated with RS. Magee equations include routinely evaluated clinicopathological features and have been shown to strongly correlate with RS (18–20, 57, 58). In this study, the regression models using the combination of the WSI-derived image features and Magee features as independent variables outperformed the models based on Magee features alone for RS correlation. The small correlation coefficients between the Magee and image features in Figure 5 and similar model coefficients in Figure 6 indicate the image features capture complementary prediction values for RS prediction. These results suggest that Magee features can enhance RS correlation when they are jointly used with the phenotypic information from WSIs.

In contrast with the substantial prediction improvement for validation set 1, a marginal improvement with validation set 2 is noticed. In Table 5, the adjusted *R*^2^ is 0.3048 and 0.0139 when the model trained with image features alone is applied to validation sets 1 and 2, respectively. This suggests a much stronger predictive value of image features from validation set 1 than validation set 2. One possible reason for limited success with validation set 2 is that images in validation set 2 were originally scanned at 20 × and computationally scaled to 40 × magnification. Such an inconsistent tissue scanning configuration may result in a significant downstream analysis difference accounting for a degraded prediction improvement. Additionally, we noticed from Table 3 that the average “TIL number variance” from validation set 2 is substantially less than that of the training set and validation set 1. To further investigate the individual feature impact on the prediction output, we computed the numerical product of each feature average value and its regression coefficient from the linear regression model. All such feature products are comparable across training set, validation set 1, and validation set 2, except for “TIL number variance.” Specifically, the numerical product for “TIL number variance” from validation set 2 (i.e., 0.16) is less than half of that from the other two datasets (i.e., 0.35 and 0.39 from training and validation set 1, respectively), potentially degrading prediction improvement.

Our regression model used three histopathological image features extracted from WSIs: “tumor cell number in the densest tile,” “TIL number variance,” and “tumor nuclear grade.” Tumor density is understudied in breast cancer prognosis. Tumor stroma has been shown to play an essential role in breast cancer prognosis and response to therapies (59–62). High tumor-stromal content was shown to correlate with poor prognosis in triple-negative breast cancer (62), although such correlation was not demonstrated in ER+ breast cancer. Our study showed that high tumor density was associated with high RS. The role of stroma and tumor density in ER+ breast cancer may be essential and warrants more studies. TIL is an important prognostic and predictive marker in HER2+ and triple-negative breast cancer (9, 10, 63–65). Although the role of TIL is controversial in ER+ breast cancer (64, 66), high TIL has been found to correlate with high RS (66, 67). RS is strongly correlated with the proliferative module (68). One possible explanation for such correlation is the increased tumor proliferative rate within high TIL areas or the high proliferative rate of TIL itself. TIL has been shown to correlate with a high proliferative index in breast cancer (38). Thus, both the increased tumor proliferation and lymphocyte proliferation could contribute to the positive correlation with RS. While evaluations of TILs by pathologists may have intra- and inter-observation variations (69, 70), machine learning provides the opportunity to better quantify the TIL assessment (71). Tumor nuclear grade has been shown as an important prognostic factor in breast cancer and is a component of the Nottingham tumor grade (37). Genes associated with tumor grade are part of the Breast Cancer Index and are strongly correlated with tumor prognosis in ER+ breast cancer (72, 73).

In our study, 54 cases had discordant recommendations for chemotherapy treatment by RS and the DL-based model. Some patients with RS recommendation for chemotherapy and low risk by DL-based model did not actually receive chemotherapy while others not recommended for chemotherapy by RS and had low risk by DL-based model received chemotherapy. However, none of these patients developed cancer recurrence, including local and distant recurrence. The absolute benefit from chemotherapy to prevent distant recurrence in patients with intermediate RS is < 10% (30). Although it is also possible that these patients did not benefit from chemotherapy simply by chance, it is also possible that the benefit from chemotherapy in these patients with discordant results is not clear, and further studies are needed.

In this study, we trained three DL models to detect the tumor cells, TILs, and segment tumor nuclei. These model architectures were built on the MRCNN with the multitasking ability for detection, classification, and segmentation. We found that the performance of a comprehensive model was often inferior to that of individual single-task models. When a model was trained with one task at a time, the same DL model could achieve better accuracy due to more focused learning of one data distribution. In contrast, the multitask DL model’s performance may deteriorate due to the high heterogeneity across multiple training sets. In our study, for instance, the circle labels for the detection model were significantly different from the mask labels for the segmentation model. The heterogeneity between the two types of data undermined the model’s learning ability after merging them as one training dataset. Therefore, we trained three individual DL models. Due to the TIL training data heterogeneity, the TIL detection model might recognize some tumor nuclei as TILs by mistake. As the public MonuSeg-2018 dataset did not include cell type labels, we found that the tumor nuclei segmentation model predicted contours of non-tumor cells. To address these issues, we used tumor nucleus detection results to remove TILs and tumor nuclei false positive. Based on the density maps from the DL predictions, we observed that tissue regions of high TIL density were close to high tumor cell density regions, as shown in Figure 4. Such proximity of these two regions was frequently observed at the tumor invading fronts, consistent with previous studies (10, 64, 74–76).

As the patient cohorts for this study were not from a prospective clinical trial, we planned to validate our findings in completed prospective clinical trials in the following work. We also planned to increase our testing patient cohorts. Although we included 382 patients in the training and validation sets, a more extensive study is needed to validate our findings.

Overall, our results suggest that the combination of the image features derived from WSIs and Magee features presents a stronger correlation with RS than the Magee features alone. Although WSI image features present complementary information for RS correlation, we do not intend to replace Magee features with these WSI image features. Instead, we proposed to further boost Magee feature performance on RS correlation with these histology features from WSIs only available after computational analysis. To the best of our knowledge, our proposed approach is innovative in the sense that it uses the histological image features from WSIs to enhance the correlation between the Magee features and RS. The Magee equations can save healthcare costs and effectively serve patients with early breast cancer (77). The DL-based processing method presented in this study can be executed automatically at high throughput and further enhance the predictive power of Magee features.

## Conclusion

In this study, we have developed a DL-based digital pathology image processing pipeline to enhance the RS correlation with histology features derived from WSIs of ER+/HER2-/LN- breast cancer tissues. The proposed DL-based pipeline accurately detected tumor cells and TILs, segmented tumor cells, and extracted histology image features from gigapixel WSIs with high throughput. We demonstrated that the image features derived by DL-based analysis enhanced Magee feature correlation with RS.

## Data Availability Statement

The original contributions presented in this study are included in the article/Supplementary Material, further inquiries can be directed to the corresponding author/s.

## Ethics Statement

The studies involving human participants were reviewed and approved by Emory University Institutional Review Board. The patients/participants provided their written informed consent to participate in this study.

## Author Contributions

HL, JK, and XL conceived the original idea and designed the research. HL and JK performed the research. JW, ZL, MD, PZ, GS, and XL contributed data collection and image annotations. HL and ML provided statistical support. HL and JK worked on the manuscript with support from FW, GT, and XL. All authors were involved in data analysis and read and approved the final manuscript.

## Conflict of Interest

The authors declare that the research was conducted in the absence of any commercial or financial relationships that could be construed as a potential conflict of interest.

## Publisher’s Note

All claims expressed in this article are solely those of the authors and do not necessarily represent those of their affiliated organizations, or those of the publisher, the editors and the reviewers. Any product that may be evaluated in this article, or claim that may be made by its manufacturer, is not guaranteed or endorsed by the publisher.

## Abbreviations

DFS: disease-free survival
DL: deep learning
ER: estrogen receptor
HD: Hausdorff distance
H&E: hematoxylin and eosin
IOU: intersection over union
MRCNN: mask region-based convolutional neural network
OS: overall survival
PR: progesterone receptor
RS: Oncotype DX Recurrence Score
TIL: tumor-infiltrating lymphocytes
WSI: whole-slide image.

## References

[1] Arciero CA Guo Y Jiang R Behera M O’Regan R Peng L ER^+^/HER2^+^ breast cancer has different metastatic patterns and better survival than ER^–^/HER2^+^ breast cancer. *Clin Breast Cancer.* (2019) 19:236–45. 10.1016/j.clbc.2019.02.001 30846407

[2] Bhattarai S Klimov S Mittal K Krishnamurti U Li XB Oprea-Ilies G Prognostic role of androgen receptor in triple negative breast cancer: a multi-institutional study. *Cancers (Basel).* (2019) 11:995. 10.3390/cancers11070995 31319547 PMC6678933

[3] Guo Y Arciero CA Jiang R Behera M Peng L Li X. Different breast cancer subtypes show different metastatic patterns: a study from a large public database. *Asian Pac J Cancer Prev.* (2020) 21:3587–93. 10.31557/APJCP.2020.21.12.3587 33369456 PMC8046324

[4] Li X Yang J Krishnamurti U Huo L Ward KC O’Regan R Hormone receptor-positive breast cancer has a worse prognosis in male than in female patients. *Clin Breast Cancer.* (2017) 17:356–66. 10.1016/j.clbc.2017.03.005 28576631

[5] Li X Zhang Y Meisel J Jiang R Behera M Peng L. Validation of the newly proposed American joint committee on cancer (AJCC) breast cancer prognostic staging group and proposing a new staging system using the national cancer database. *Breast Cancer Res Treat.* (2018) 171:303–13. 10.1007/s10549-018-4832-9 29948405

[6] Reddy TP Rosato RR Li X Moulder S Piwnica-Worms H Chang JC. A comprehensive overview of metaplastic breast cancer: clinical features and molecular aberrations. *Breast Cancer Res.* (2020) 22:121. 10.1186/s13058-020-01353-z 33148288 PMC7640663

[7] Zhao J Krishnamurti U Zhang C Meisel J Wei Z Suo A HER2 immunohistochemistry staining positivity is strongly predictive of tumor response to neoadjuvant chemotherapy in HER2 positive breast cancer. *Pathol Res Pract.* (2020) 216:153155. 10.1016/j.prp.2020.153155 32871536

[8] Li X Oprea-Ilies GM Krishnamurti U. New developments in breast cancer and their impact on daily practice in pathology. *Arch Pathol Lab Med.* (2017) 141:490–8. 10.5858/arpa.2016-0288-SA 28353377

[9] Meisel JL Zhao J Suo A Zhang C Wei Z Taylor C Clinicopathologic factors associated with response to neoadjuvant anti-HER2-directed chemotherapy in HER2-positive breast cancer. *Clin Breast Cancer.* (2020) 20:19–24. 10.1016/j.clbc.2019.09.003 31806448

[10] Zhao J Meisel J Guo Y Nahta R Hsieh KL Peng L Evaluation of PD-L1, tumor-infiltrating lymphocytes, and CD8^+^ and FOXP3^+^ immune cells in HER2-positive breast cancer treated with neoadjuvant therapies. *Breast Cancer Res Treat.* (2020) 183:599–606. 10.1007/s10549-020-05819-8 32715443

[11] Eifel P Axelson JA Costa J Crowley J Curran WJ Jr. Deshler A National institutes of health consensus development conference statement: adjuvant therapy for breast cancer, november 1-3, 2000. *J Natl Cancer Inst.* (2001) 93:979–89. 11438563 10.1093/jnci/93.13.979

[12] Goldhirsch A Wood WC Gelber RD Coates AS Thurlimann B Senn HJ. Meeting highlights: updated international expert consensus on the primary therapy of early breast cancer. *J Clin Oncol.* (2003) 21:3357–65. 10.1200/JCO.2003.04.576 12847142

[13] Sparano JA Paik S. Development of the 21-gene assay and its application in clinical practice and clinical trials. *J Clin Oncol.* (2008) 26:721–8. 10.1200/JCO.2007.15.1068 18258979

[14] Paik S Tang G Shak S Kim C Baker J Kim W Gene expression and benefit of chemotherapy in women with node-negative, estrogen receptor-positive breast cancer. *J Clin Oncol.* (2006) 24:3726–34. 10.1200/JCO.2005.04.7985 16720680

[15] Harris LN Ismaila N McShane LM Andre F Collyar DE Gonzalez-Angulo AM Use of biomarkers to guide decisions on adjuvant systemic therapy for women with early-stage invasive breast cancer: American society of clinical oncology clinical practice guideline. *J Clin Oncol.* (2016) 34:1134–50. 26858339 10.1200/JCO.2015.65.2289 PMC4933134

[16] Sparano JA Gray RJ Makower DF Pritchard KI Albain KS Hayes DF Prospective validation of a 21-gene expression assay in breast cancer. *N Engl J Med.* (2015) 373:2005–14. 10.1056/NEJMoa1510764 26412349 PMC4701034

[17] Kalinsky K Barlow WE Meric-Bernstam F Gralow JR Albain KS Hayes D First results from a phase III randomized clinical trial of standard adjuvant endocrine therapy (ET) +/- chemotherapy (CT) in patients (pts) with 1–3 positive nodes, hormone receptor-positive (HR+) and HER2-negative (HER2-) breast cancer (BC) with recurrence score (RS) < 25: SWOG S1007 (RxPonder). *Cancer Res.* (2021) 81. 10.1158/1538-7445.SABCS20-GS3-00

[18] Bhargava R Dabbs DJ. Magee equations and oncotype DX(^®^)-a perspective. *Breast Cancer Res Treat.* (2017) 164:245–6. 10.1007/s10549-017-4235-3 28393310

[19] Bhargava R Esposito NN O’Connor SM Li Z Turner BM Moisini I Magee equations and response to neoadjuvant chemotherapy in ER^+^/HER2-negative breast cancer: a multi-institutional study. *Mod Pathol.* (2021) 34:77–84. 10.1038/s41379-020-0620-2 32661297

[20] Flanagan MB Dabbs DJ Brufsky AM Beriwal S Bhargava R. Histopathologic variables predict oncotype DX recurrence score. *Mod Pathol.* (2008) 21:1255–61. 10.1038/modpathol.2008.54 18360352

[21] Roy M Wang F Vo H Teng D Teodoro G Farris AB Deep-learning-based accurate hepatic steatosis quantification for histological assessment of liver biopsies. *Lab Invest.* (2020) 100:1367–83. 10.1038/s41374-020-0463-y 32661341 PMC7502534

[22] Yokoyama S Hamada T Higashi M Matsuo K Maemura K Kurahara H Predicted prognosis of patients with pancreatic cancer by machine learning. *Clin Cancer Res.* (2020) 26:2411–21. 31992588 10.1158/1078-0432.CCR-19-1247

[23] Jaber MI Song B Taylor C Vaske CJ Benz SC Rabizadeh S A deep learning image-based intrinsic molecular subtype classifier of breast tumors reveals tumor heterogeneity that may affect survival. *Breast Cancer Res.* (2020) 22:12. 10.1186/s13058-020-1248-3 31992350 PMC6988279

[24] Liu Y Kohlberger T Norouzi M Dahl GE Smith JL Mohtashamian A Artificial intelligence-based breast cancer nodal metastasis detection: insights into the black box for pathologists. *Arch Pathol Lab Med.* (2019) 143:859–68. 10.5858/arpa.2018-0147-OA 30295070

[25] Pantanowitz L Quiroga-Garza GM Bien L Heled R Laifenfeld D Linhart C An artificial intelligence algorithm for prostate cancer diagnosis in whole slide images of core needle biopsies: a blinded clinical validation and deployment study. *Lancet Digit Health.* (2020) 2:e407–16. 10.1016/S2589-7500(20)30159-X 33328045

[26] Jiang Y Yang M Wang S Li X Sun Y. Emerging role of deep learning-based artificial intelligence in tumor pathology. *Cancer Commun (Lond).* (2020) 40:154–66. 10.1002/cac2.12012 32277744 PMC7170661

[27] Cheplygina V de Bruijne M Pluim JPW. Not-so-supervised: a survey of semi-supervised, multi-instance, and transfer learning in medical image analysis. *Med Image Anal.* (2019) 54:280–96. 10.1016/j.media.2019.03.009 30959445

[28] Shen D Wu G Suk HI. Deep learning in medical image analysis. *Annu Rev Biomed Eng.* (2017) 19:221–48. 28301734 10.1146/annurev-bioeng-071516-044442 PMC5479722

[29] Litjens G Kooi T Bejnordi BE Setio AAA Ciompi F Ghafoorian M A survey on deep learning in medical image analysis. *Med Image Anal.* (2017) 42:60–88. 28778026 10.1016/j.media.2017.07.005

[30] Sparano JA Gray RJ Ravdin PM Makower DF Pritchard KI Albain KS Clinical and genomic risk to guide the use of adjuvant therapy for breast cancer. *N Engl J Med.* (2019) 380:2395–405. 10.1056/NEJMoa1904819 31157962 PMC6709671

[31] Allison KH Hammond MEH Dowsett M McKernin SE Carey LA Fitzgibbons PL Estrogen and progesterone receptor testing in breast cancer: ASCO/CAP guideline update. *J Clin Oncol.* (2020) 38:1346–66. 10.1200/JCO.19.02309 31928404

[32] Wolff AC Hammond MEH Allison KH Harvey BE Mangu PB Bartlett JMS Human epidermal growth factor receptor 2 testing in breast cancer: American society of clinical oncology/college of American pathologists clinical practice guideline focused update. *J Clin Oncol.* (2018) 36:2105–22. 29846122 10.1200/JCO.2018.77.8738

[33] Vahadane A Peng T Sethi A Albarqouni S Wang L Baust M Structure-preserving color normalization and sparse stain separation for histological images. *IEEE Trans Med Imaging.* (2016) 35:1962–71. 10.1109/TMI.2016.2529665 27164577

[34] He K Gkioxari G Dollár P Girshick R. Mask R-CNN. In: *Proceedings of the IEEE International Conference on Computer Vision.* Venice (2017). p. 2961–9.

[35] Ren S He K Girshick R Sun J. Faster R-CNN: towards real-time object detection with region proposal networks. *IEEE Trans Pattern Anal Mach Intell.* (2017) 39:1137–49. 10.1109/TPAMI.2016.2577031 27295650

[36] Girshick R. Fast R-CNN. In: *Proceedings of the IEEE International Conference on Computer Vision.* Santiago (2015). p. 1440–8. 10.1109/ICCV.2015.169

[37] Kerlikowske K Molinaro A Cha I Ljung BM Ernster VL Stewart K Characteristics associated with recurrence among women with ductal carcinoma in situ treated by lumpectomy. *J Natl Cancer Inst.* (2003) 95:1692–702. 10.1093/jnci/djg097 14625260

[38] Bouzidi L Triki H Charfi S Kridis WB Derbel M Ayadi L Prognostic value of natural killer cells besides tumor-infiltrating lymphocytes in breast cancer tissues. *Clin Breast Cancer.* (2021) 21:e738–47. 10.1016/j.clbc.2021.02.003 33727019

[39] Klein ME Dabbs DJ Shuai Y Brufsky AM Jankowitz R Puhalla SL Prediction of the oncotype DX recurrence score: use of pathology-generated equations derived by linear regression analysis. *Mod Pathol.* (2013) 26:658–64. 10.1038/modpathol.2013.36 23503643 PMC3647116

[40] Geradts J Bean SM Bentley RC Barry WT. The oncotype DX recurrence score is correlated with a composite index including routinely reported pathobiologic features. *Cancer Invest.* (2010) 28:969–77. 10.3109/07357907.2010.512600 20873988

[41] Tang P Wang J Hicks DG Wang X Schiffhauer L McMahon L A lower allred score for progesterone receptor is strongly associated with a higher recurrence score of 21-gene assay in breast cancer. *Cancer Invest.* (2010) 28:978–82. 10.3109/07357907.2010.496754 20690804

[42] Cuzick J Dowsett M Pineda S Wale C Salter J Quinn E Prognostic value of a combined estrogen receptor, progesterone receptor, Ki-67, and human epidermal growth factor receptor 2 immunohistochemical score and comparison with the genomic health recurrence score in early breast cancer. *J Clin Oncol.* (2011) 29:4273–8. 10.1200/JCO.2010.31.2835 21990413

[43] Eaton AA Pesce CE Murphy JO Stempel MM Patil SM Brogi E Estimating the OncotypeDX score: validation of an inexpensive estimation tool. *Breast Cancer Res Treat.* (2017) 161:435–41. 10.1007/s10549-016-4069-4 27928699 PMC5310948

[44] Allison KH Kandalaft PL Sitlani CM Dintzis SM Gown AM. Routine pathologic parameters can predict oncotype DXTM recurrence scores in subsets of ER positive patients: who does not always need testing? *Breast Cancer Res Treat.* (2012) 131:413–24. 10.1007/s10549-011-1416-3 21369717

[45] Ingoldsby H Webber M Wall D Scarrott C Newell J Callagy G. Prediction of oncotype DX and TAILORx risk categories using histopathological and immunohistochemical markers by classification and regression tree (CART) analysis. *Breast.* (2013) 22:879–86. 10.1016/j.breast.2013.04.008 23643806

[46] Kim H-S Umbricht CB Illei PB Cimino-Mathews A Cho S Chowdhury N Optimizing the use of gene expression profiling in early-stage breast cancer. *J Clin Oncol.* (2016) 34:4390–7. 10.1200/JCO.2016.67.7195 27998227 PMC5455310

[47] Orucevic A Bell JL McNabb AP Heidel RE. Oncotype DX breast cancer recurrence score can be predicted with a novel nomogram using clinicopathologic data. *Breast Cancer Res Treat.* (2017) 163:51–61. 10.1007/s10549-017-4170-3 28243897 PMC5387031

[48] Hanna MG Bleiweiss IJ Nayak A Jaffer S. Correlation of oncotype DX recurrence score with Histomorphology and Immunohistochemistry in over 500 patients. *Int J Breast Cancer.* (2017) 2017:1257078. 10.1155/2017/1257078 28168058 PMC5266836

[49] Lee SB Kim J Sohn G Kim J Chung IY Kim HJ A nomogram for predicting the oncotype DX recurrence score in women with T1-3N0-1miM0 hormone receptor?positive, human epidermal growth factor 2 (HER2) negative breast cancer. *Cancer Res Treat.* (2019) 51:1073–85. 10.4143/crt.2018.357 30384581 PMC6639212

[50] Wu S-G Zhang W-W Wang J Lian C-L Sun J-Y Chen Y-X Progesterone receptor status and tumor grade predict the 21-gene recurrence score of invasive lobular breast cancer. *Biomark Med.* (2019) 13:1005–12. 10.2217/bmm-2019-0209 31234641

[51] Thibodeau S Voutsadakis IA. Prediction of oncotype DX recurrence score using clinical parameters: a comparison of available tools and a simple predictor based on grade and progesterone receptor. *Hematol Oncol Stem Cell Ther.* (2019) 12:89–96. 10.1016/j.hemonc.2019.02.001 30796885

[52] Orucevic A Bell JL King M McNabb AP Heidel RE. Nomogram update based on TAILORx clinical trial results – oncotype DX breast cancer recurrence score can be predicted using clinicopathologic data. *Breast.* (2019) 46:116–25. 10.1016/j.breast.2019.05.006 31146185

[53] Baltres A Al Masry Z Zemouri R Valmary-Degano S Arnould L Zerhouni N Prediction of oncotype DX recurrence score using deep multi-layer perceptrons in estrogen receptor-positive, HER2-negative breast cancer. *Breast Cancer.* (2020) 27:1007–16. 10.1007/s12282-020-01100-4 32385567

[54] Yepes MM Romilly AP Collado-Mesa F Net JM Kiszonas R Arheart KL Can mammographic and sonographic imaging features predict the oncotype DX™ recurrence score in T1 and T2, hormone receptor positive, HER2 negative and axillary lymph node negative breast cancers? *Breast Cancer Res Treat.* (2014) 148:117–23. 10.1007/s10549-014-3143-z 25262341

[55] Saha A Harowicz MR Wang W Mazurowski MA. A study of association of oncotype DX recurrence score with DCE-MRI characteristics using multivariate machine learning models. *J Cancer Res Clin Oncol.* (2018) 144:799–807. 10.1007/s00432-018-2595-7 29427210 PMC5920720

[56] Ha R Chang P Mutasa S Karcich J Goodman S Blum E Convolutional neural network using a breast MRI tumor dataset can predict oncotype Dx recurrence score. *J Magn Reson Imagings.* (2019) 49:518–24. 10.1002/jmri.26244 30129697 PMC8139130

[57] Hou Y Zynger DL Li X Li Z. Comparison of oncotype DX with modified magee equation recurrence scores in low-grade invasive carcinoma of breast. *Am J Clin Pathol.* (2017) 148:167–72. 10.1093/ajcp/aqx059 28898988

[58] Hou Y Moosavi HS Wei L Parwani AV Li XB Li Z. Magee equation recurrence score is associated with distal metastatic risk in male breast carcinomas: experience from two institutions. *Am J Clin Pathol.* (2018) 150:491–8. 10.1093/ajcp/aqy078 30084931

[59] Plava J Cihova M Burikova M Matuskova M Kucerova L Miklikova S. Recent advances in understanding tumor stroma-mediated chemoresistance in breast cancer. *Mol Cancer.* (2019) 18:67. 10.1186/s12943-019-0960-z 30927930 PMC6441200

[60] Bussard KM Mutkus L Stumpf K Gomez-Manzano C Marini FC. Tumor-associated stromal cells as key contributors to the tumor microenvironment. *Breast Cancer Res.* (2016) 18:84. 10.1186/s13058-016-0740-2 27515302 PMC4982339

[61] Hill BS Sarnella A D’Avino G Zannetti A. Recruitment of stromal cells into tumour microenvironment promote the metastatic spread of breast cancer. *Semin Cancer Biol.* (2020) 60:202–13. 10.1016/j.semcancer.2019.07.028 31377307

[62] Kramer CJH Vangangelt KMH van Pelt GW Dekker TJA Tollenaar R Mesker WE. The prognostic value of tumour-stroma ratio in primary breast cancer with special attention to triple-negative tumours: a review. *Breast Cancer Res Treat.* (2019) 173:55–64. 10.1007/s10549-018-4987-4 30302588 PMC6394568

[63] Li XB Krishnamurti U Bhattarai S Klimov S Reid MD O’Regan R Biomarkers predicting pathologic complete response to neoadjuvant chemotherapy in breast cancer. *Am J Clin Pathol.* (2016) 145:871–8. 10.1093/ajcp/aqw045 27298399

[64] Krishnamurti U Wetherilt CS Yang J Peng L Li X. Tumor-infiltrating lymphocytes are significantly associated with better overall survival and disease-free survival in triple-negative but not estrogen receptor-positive breast cancers. *Hum Pathol.* (2017) 64:7–12. 10.1016/j.humpath.2017.01.004 28153508

[65] Gonzalez-Ericsson PI Stovgaard ES Sua LF Reisenbichler E Kos Z Carter JM The path to a better biomarker: application of a risk management framework for the implementation of PD-L1 and TILs as immuno-oncology biomarkers in breast cancer clinical trials and daily practice. *J Pathol.* (2020) 250:667–84. 10.1002/path.5406 32129476

[66] Kolberg-Liedtke C Gluz O Heinisch F Feuerhake F Kreipe H Clemens M Association of TILs with clinical parameters, recurrence score^®^ results, and prognosis in patients with early HER2-negative breast cancer (BC)-a translational analysis of the prospective WSG PlanB trial. *Breast Cancer Res.* (2020) 22:47. 10.1186/s13058-020-01283-w 32408905 PMC7227091

[67] Ahn SG Cha YJ Bae SJ Yoon C Lee HW Jeong J. Comparisons of tumor-infiltrating lymphocyte levels and the 21-gene recurrence score in ER-positive/HER2-negative breast cancer. *BMC Cancer.* (2018) 18:320. 10.1186/s12885-018-4228-6 29573739 PMC5866511

[68] Buus R Sestak I Kronenwett R Ferree S Schnabel CA Baehner FL Molecular drivers of oncotype DX, prosigna, ENDOPREDICT, and the breast cancer index: a TransATAC study. *J Clin Oncol.* (2021) 39:126–35. 10.1200/JCO.20.00853 33108242 PMC8078458

[69] Swisher SK Wu Y Castaneda CA Lyons GR Yang F Tapia C Interobserver agreement between pathologists assessing tumor-infiltrating lymphocytes (TILs) in breast cancer using methodology proposed by the international TILs working group. *Ann Surg Oncol.* (2016) 23:2242–8. 10.1245/s10434-016-5173-8 26965699

[70] O’Loughlin M Andreu X Bianchi S Chemielik E Cordoba A Cserni G Reproducibility and predictive value of scoring stromal tumour infiltrating lymphocytes in triple-negative breast cancer: a multi-institutional study. *Breast Cancer Res Treat.* (2018) 171:1–9. 10.1007/s10549-018-4825-8 29774470

[71] Klauschen F Müller KR Binder A Bockmayr M Hägele M Seegerer P Scoring of tumor-infiltrating lymphocytes: from visual estimation to machine learning. *Semin Cancer Biol.* (2018) 52:151–7. 10.1016/j.semcancer.2018.07.001 29990622

[72] Zhang Y Schnabel CA Schroeder BE Jerevall PL Jankowitz RC Fornander T Breast cancer index identifies early-stage estrogen receptor-positive breast cancer patients at risk for early- and late-distant recurrence. *Clin Cancer Res.* (2013) 19:4196–205. 10.1158/1078-0432.CCR-13-0804 23757354

[73] Sgroi DC Sestak I Cuzick J Zhang Y Schnabel CA Schroeder B Prediction of late distant recurrence in patients with oestrogen-receptor-positive breast cancer: a prospective comparison of the breast-cancer index (BCI) assay, 21-gene recurrence score, and IHC4 in the TransATAC study population. *Lancet Oncol.* (2013) 14:1067–76. 10.1016/S1470-2045(13)70387-5 24035531 PMC3918681

[74] Buisseret L Garaud S de Wind A Van den Eynden G Boisson A Solinas C Tumor-infiltrating lymphocyte composition, organization and PD-1/PD-L1 expression are linked in breast cancer. *Oncoimmunology.* (2017) 6:e1257452. 10.1080/2162402X.2016.1257452 28197375 PMC5283629

[75] Le H Gupta R Hou L Abousamra S Fassler D Torre-Healy L Utilizing automated breast cancer detection to identify spatial distributions of tumor-infiltrating lymphocytes in invasive breast cancer. *Am J Pathol.* (2020) 190:1491–504. 10.1016/j.ajpath.2020.03.012 32277893 PMC7369575

[76] Li X Wetherilt CS Krishnamurti U Yang J Ma Y Styblo TM Stromal PD-L1 expression is associated with better disease-free survival in triple-negative breast cancer. *Am J Clin Pathol.* (2016) 146:496–502. 10.1093/ajcp/aqw134 27686176

[77] Bhargava R Clark BZ Carter GJ Brufsky AM Dabbs DJ. The healthcare value of the magee decision algorithm™: use of magee equations™ and mitosis score to safely forgo molecular testing in breast cancer. *Mod Pathol.* (2020) 33:1563–70. 10.1038/s41379-020-0521-4 32203092 PMC7384988

