# Supplementary material for: Deep Learning-Based Pathology Image Analysis Enhances Magee Feature Correlation With Oncotype DX Breast Recurrence Score
Source: Front Med (Lausanne). 2022 Jun 14;9:886763. doi: 10.3389/fmed.2022.886763 (PMC9239530; doi:10.3389/fmed.2022.886763)
Supplement: Supplementary file 1 [file Data_Sheet_1.pdf]

10.3389/fmed.2022.886763

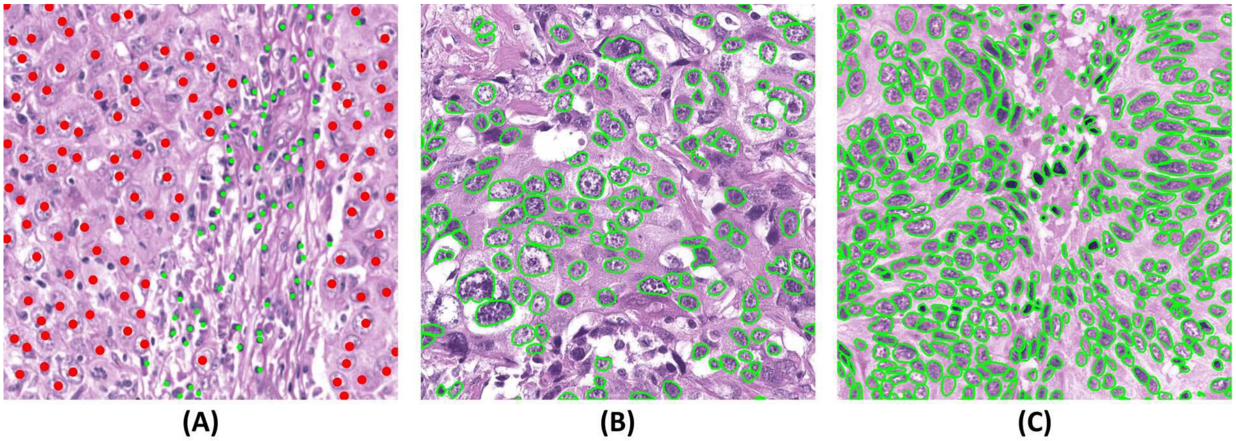

Supplementary Figure 1. Examples of (A) point annotations for tumor cells and TILs, (B) contour annotations for tumor nuclei from an independent dataset, and (C) contour annotations for tumor nuclei from the MoNuSeg-2018 dataset. In (A), point annotations for tumor cells and TILs are red and green, respectively. In (B) and (C), annotated tumor nuclei boundaries are represented by green contours.

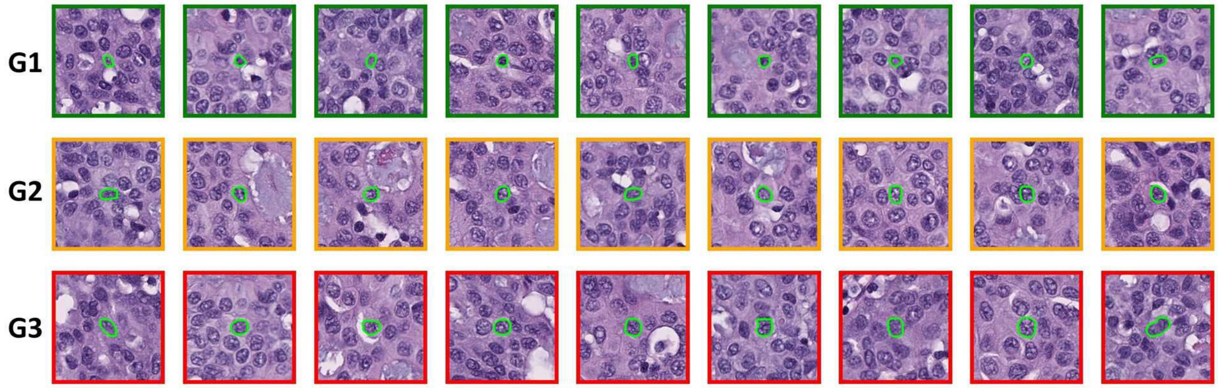

Supplementary Figure 2. Demonstrations of tumor cells of different nuclear grades. After tumor nuclei are segmented, each nucleus is assigned one nuclear grade determined by the ratio of the tumor nucleus size to the "standard" TIL size. The nuclear grade (G) is defined as 1, 2, and 3 when such ratio is between 1 and 2.5, 2.5 and 3.5, and greater than 3.5, respectively. The images are arranged by the ascending ratio order in each row.

10.3389/fmed.2022.886763

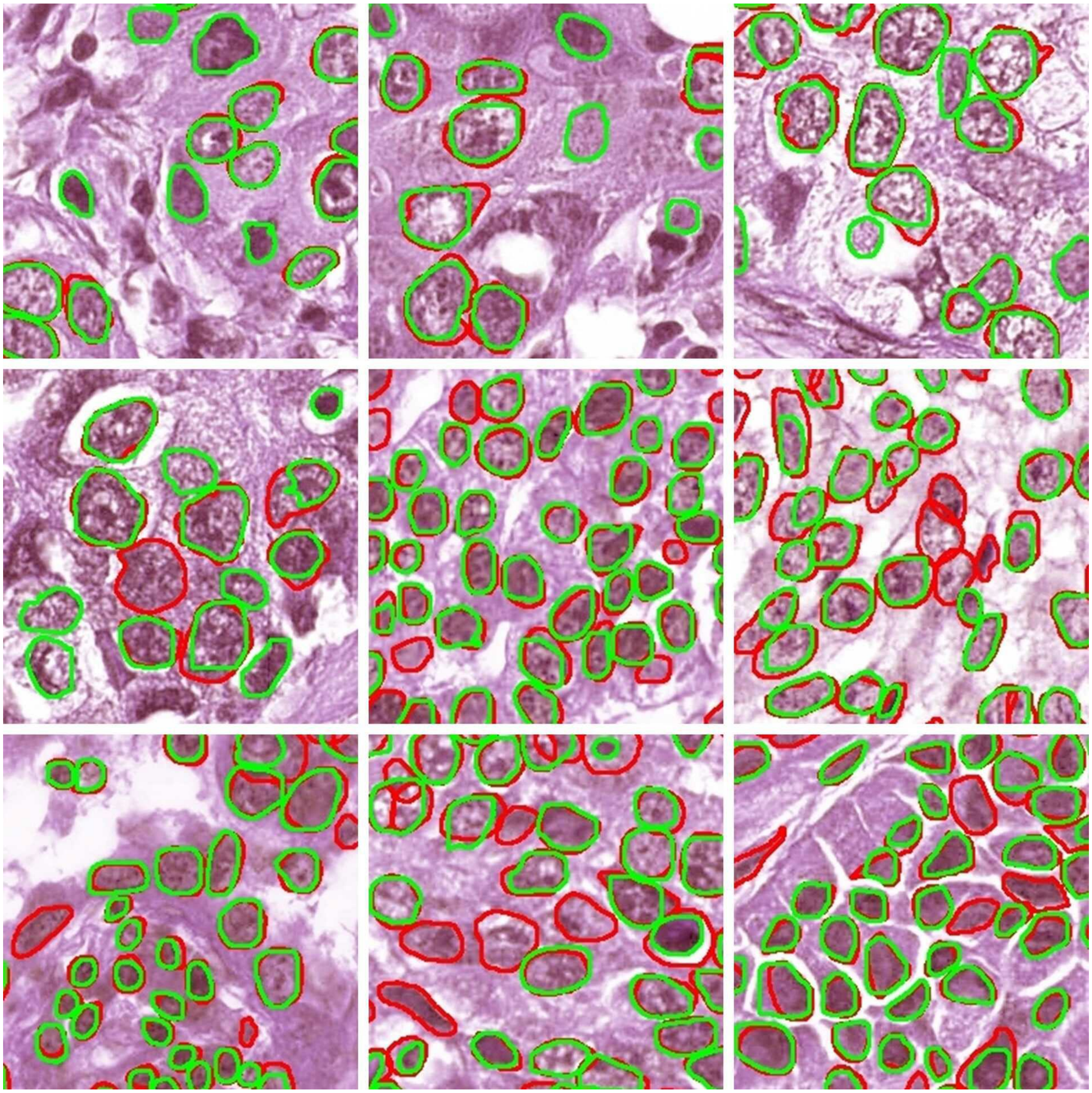

Supplementary Figure 3. Example of the ground truths and predictions of segmentation. The red contours are the ground truths annotated by pathologists, and the green ones are the predictions.

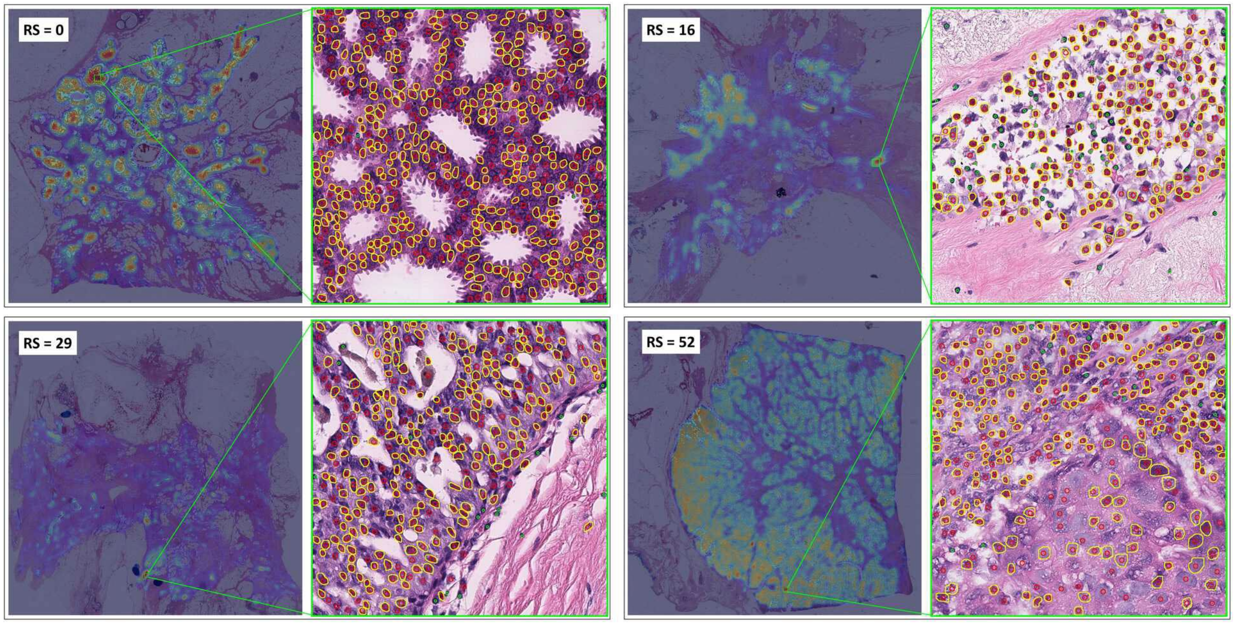

Supplementary Figure 4. Four representative cases were selected from Validation Set 1. In each case, the left is the WSI tumor cell density map, and the right is the tumor-highest-density region with predictions. The detected TIL and tumor cell nuclei are indicated by green and red circles, respectively. The predicted contours of the tumor cell nuclei are indicated in yellow.

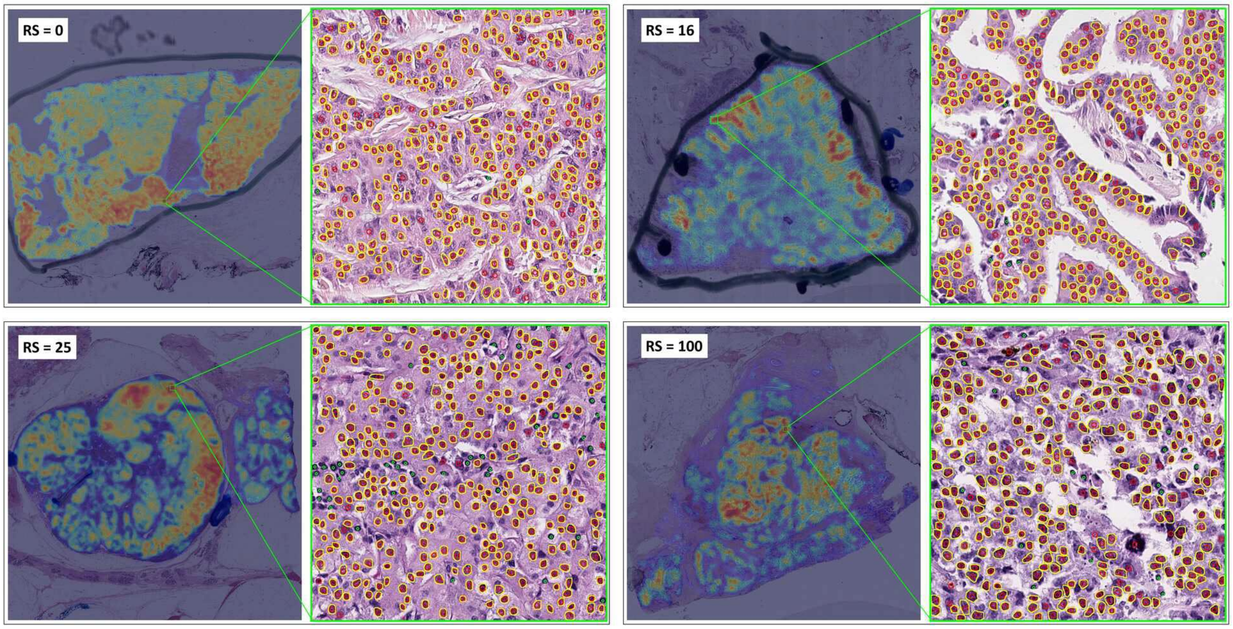

Supplementary Figure 5. Four representative cases were selected from Validation Set 2. In each case, the left is the WSI tumor cell density map, and the right is the tumor-highest-density region with predictions. The detected TIL and tumor cell nuclei are indicated by green and red circles, respectively. The predicted contours of the tumor cell nuclei are indicated in yellow.

10.3389/fmed.2022.886763

Supplementary Table 1. Summary of the detected cell numbers from WSIs in three cohort datasets.

	Tumor number			TIL number		
	Minimum	Median	Maximum	Minimum	Median	Maximum
Training Set	4,295	102,902	733,937	2,649	34,748	708,913
Validation Set 1	2,430	80,334	417,601	1,135	42,943	268,778
Validation Set 2	1,206	54,440	382,172	228	27,276	390,641
